# Improved Real-Time Facial Expression Recognition Based on a Novel Balanced and Symmetric Local Gradient Coding

**DOI:** 10.3390/s19081899

**Published:** 2019-04-22

**Authors:** Jucheng Yang, Xiaojing Wang, Shujie Han, Jie Wang, Dong Sun Park, Yuan Wang

**Affiliations:** 1 College of Computer Science and Information Engineering, Tianjin University of Science and Technology, Tianjin 300457, China; wxj@tust.edu.cn (X.W.); ShujieHan@mail.tust.edu.cn (S.H.); wjie@mail.tust.edu.cn (J.W.); wangyuan23@tust.edu.cn (Y.W.); 2 Department of Electronic and Information Engineering, Chonbuk National University, Jeonbuk 561-756, Korea

**Keywords:** face expression recognition, local gradient coding, feature extraction, central symmetric local gradient coding, extreme learning machine

## Abstract

In the field of Facial Expression Recognition (FER), traditional local texture coding methods have a low computational complexity, while providing a robust solution with respect to occlusion, illumination, and other factors. However, there is still need for improving the accuracy of these methods while maintaining their real-time nature and low computational complexity. In this paper, we propose a feature-based FER system with a novel local texture coding operator, named central symmetric local gradient coding (CS-LGC), to enhance the performance of real-time systems. It uses four different directional gradients on 5 × 5 grids, and the gradient is computed in the center-symmetric way. The averages of the gradients are used to reduce the sensitivity to noise. These characteristics lead to symmetric of features by the CS-LGC operator, thus providing a better generalization capability in comparison to existing local gradient coding (LGC) variants. The proposed system further transforms the extracted features into an eigen-space using a principal component analysis (PCA) for better representation and less computation; it estimates the intended classes by training an extreme learning machine. The recognition rate for the JAFFE database is 95.24%, whereas that for the CK+ database is 98.33%. The results show that the system has advantages over the existing local texture coding methods.

## 1. Introduction

Facial expressions include a wealth of information, which directly reflects people’s psychological characteristics, providing a very important means of communication. Psychologists have reported that communicating emotional information consists of approximately 7% of the language used, 38% of the tone used, and 55% of the facial expressions used [1]. It can be seen, in the course of human communication, if they cannot communicate with language, they can express basic emotions through their facial expressions. Therefore, FER has been widely studied and has become a research hotspot [2].

In addition to its relevance in human to human communication, advanced intelligent interactions between humans and machines is also an important goal driving the study of FER [3]. At present, FER systems are widely used in medical, education, driving [4] and other fields. Especially because facial expressions contain abundant psychological information, this work could be assistive to both researchers and the broader community applying FER methods.

FER system includes expression image preprocessing, face detection, region segmentation, expression feature extraction, classification, and other components. Expression feature extraction is essential for FER systems [5]. Various feature extraction algorithms, based on the local feature extraction methods, provide strong stability to light and changes of the scene orientation. Local binary pattern (LBP) [6] calculates the order of gradient between each pixel in the image and its local neighborhood points. Then the binary order relation is coded to form a local binary pattern. Finally, the multi-region histogram is used as the feature description of the image. Jabid and Chae [7] further improved the LBP algorithm to Local Directional Pattern (LDP), which has good robustness to illumination and less computation. The Gabor wavelet [6] kernel function has the same characteristics as the two-dimensional reflector of simple cells in human cerebral cortex, which can better describe texture features. Histograms of oriented gradients (HOG) [8] is also a common feature extraction algorithm. HOG features are constructed by calculating and counting the gradient direction histogram of the local area of the image. It is robust to geometric and optical changes of image. Adaptive Neuro-fuzzy Inference Systems (ANFIS) [9] is an image compression prediction coding algorithm for constructing predictors, which is also used in the field of facial expression recognition. In addition, there are local Gabor binary patterns (LGBP) [10], scale-invariant feature transform (SIFT) [11] and some corresponding improved algorithms [12]. These have been widely used in face recognition, texture classification, facial expression recognition, and other fields. These traditional methods tend to be more effective in specific small sample sets. In recent time, deep learning has become very useful for many computer vision applications [13,14]. However, due to the large amount of data required for deep learning as well as long training time, a traditional algorithm is selected for further improvement. In local feature operators, LBP operator and Gabor wavelet transform are representative methods. However, Gabor wavelet transform takes a long time and has a large feature dimension. The method of reducing the dimension will affect the accuracy of facial expression. The LBP operator and some improved methods are not perfect in facial expression recognition. These operators are unable to accurately describe texture features such as facial muscles and wrinkles. 

In order to address this problem, in 2014, Ying et al. have proposed a Local Gradient Coding (LGC) [15] algorithm. The image texture feature is extracted by encoding the strength of the neighborhood from the four gradient directions of the horizontal, vertical, and diagonal lines in the 3 × 3 neighborhood. This method fully considers the influence of texture changes on the expression and can improve the recognition rate. However, the neighborhood of the algorithm is fixed and the image texture feature has performance limitations on large scale problems. It is easy to lose part of the gradient direction information and is susceptible to noise. In order to improve this deficiency, in 2017, Shen et al. proposed an enhanced LGC facial expression recognition (LGC-FN) [16] based on a 5 × 5 neighborhood. In the 5 × 5 neighborhood, feature values are calculated in three directions, horizontal and two diagonal, around the target pixel. Finally, classification is performed using a support vector machine (SVM). This method has further improved the recognition rate.

In the neighborhood division, the LGC-FN uses the 5 × 5 neighborhood to calculate the eigenvalues, but in the calculation process, there are two key limitations. First, vertical gradient information is not considered. Although this information could be added in a simple way, it results in a higher computational dimension; this is not ideal for many real-time applications. Second, the horizontal, vertical, and diagonal information are not balanced. 

In order to solve these problems, we propose an innovative algorithm, called Central Symmetric Local Gradient Coding (CS-LGC), which defines the neighborhood as a 5 × 5 grid, uses the concept of center symmetry, and extracts the gradient information in the horizontal, vertical, and two diagonal directions. This algorithm presents several advantages. First, it extracts the gradient information in four directions as an average so that the obtained feature values of the target pixels are more representative. Second, the gradient information is more balanced and comprehensive, and the computational dimension does not increase. Finally, the algorithm has good generalization ability and can be widely used in the field of facial expression recognition. 

The rest of this paper is arranged as follows: the materials and methods for this research are presented in Section 2, where Section 2.1 introduces the basic theory of the related research methods and Section 2.2 presents our proposed method. Section 3 presents the results of experimental studies to evaluate the performance of the proposed method; an analysis of the experimental results is presented in Section 4. Section 5 presents a summary of the proposed approach and a direction for future research.

## 2. Materials and Methods

### 2.1. Related Work

#### 2.1.1. The LBP Operator Theory

**Figure 1:**
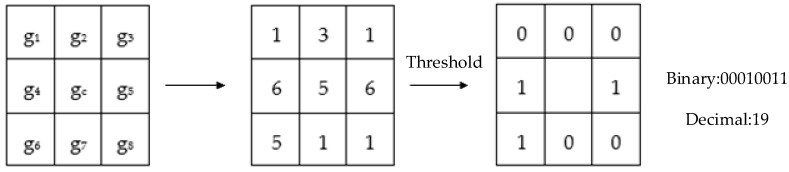
The example LBP operator.

The LBP operator is the first operator to use local features for biometric recognition. Usually, it is defined in the 3 × 3 neighborhood, and the pixel value of its center point is taken as the threshold value, which is subtracted from the surrounding points. If the result is greater than or equal 0, it is set to 1; otherwise is set to 0. Finally, the result is arranged in order to compute a binary value of the center point. This value is then converted to its decimal representation, that is, the corresponding gray value of the point. The specific process of the LBP algorithm is shown in Figure 1. The formula of the operator is shown in (1):

(1) $$\mathrm{LBP}\left(g_{c}\right)=\;\overset{p-1}{\underset{p=0}{\sum }}s\left(g_{i}-g_{c}\right)\cdot 2^{p}\;;s\left(x\right)=\{\begin{array}{c} 1\;\;x\geq 0\\ 0\;\;x<0 \end{array}$$

In the above formulas, *c* denotes the central pixel, $g_{c}$ denotes the gray value of the central pixel, $g_{i}$ denotes the gray value of the surrounding pixels, and *p* is the sampling point number. The LBP algorithm also has many derivative algorithms, such as Center-Symmetric Local Binary Patterns (CSLBP) operators. A CSLBP operator applies the idea of centrosymmetry to an LBP operator. The computational complexity of LBP operators is reduced.

#### 2.1.2. The LGC Operator Theory

**Figure 2:**
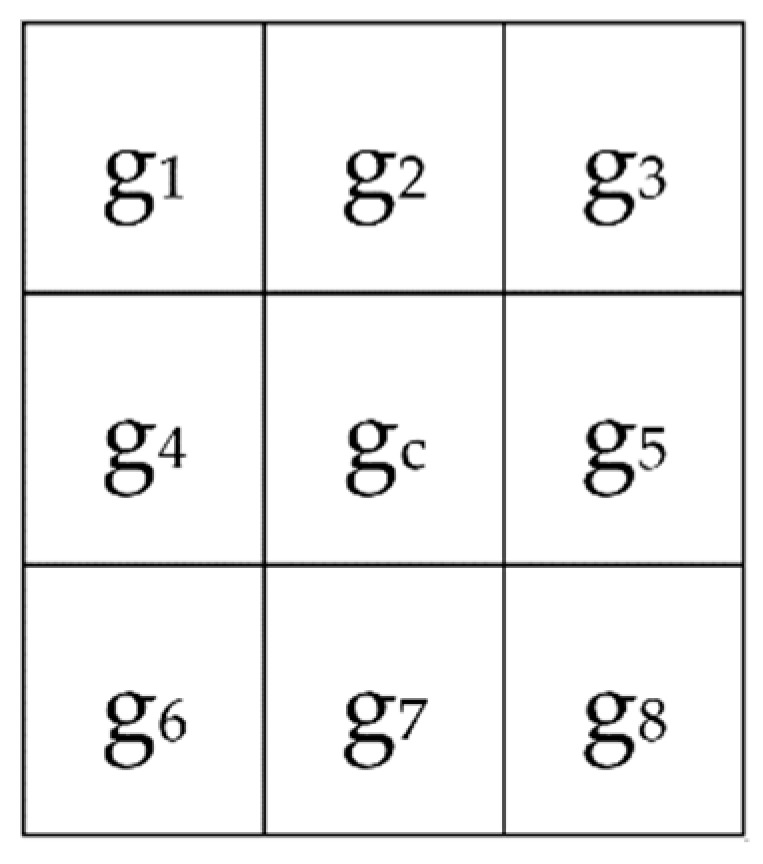
Sample mask for 3 × 3 operators.

The neighborhood size of the LGC operator is a 3 × 3 grid, as shown in Figure 2. The LGC operator compares the gray values in the horizontal, vertical, and diagonal directions for eight adjacent pixels to obtain an 8-bit binary number. The binary coding is converted into a decimal number and used to compute the LGC histogram statistics and link all of the sub-image LGC histogram features to form the entire face of identification features. The operator is defined by (2) below, where P is the sampling point number and R is the radius of the neighborhood area. Usually, R = 1, P = 8:

(2) $$\begin{array}{c} LGC_{P}^{R}=s\left(g_{1}-g_{3}\right)2^{7}+s\left(g_{4}-g_{5}\right)2^{6}+s\left(g_{6}-g_{8}\right)2^{5}+s\left(g_{1}-g_{6}\right)2^{4}+s\left(g_{2}-g_{7}\right)2^{3}\\ +s\left(g_{3}-g_{8}\right)2^{2}+s\left(g_{1}-g_{8}\right)2^{1}+s\left(g_{3}-g_{6}\right)2^{0} \end{array}$$

This encoding method is consistent with the changing trend of human eyes, mouth, forehead, and other characteristic points; in addition, it fully considers the effect of texture changes on expression. Compared with traditional LBP operators, LGC operators have stronger discrimination capabilities.

LGC operators also have some extension operators, such as LGC-HD (LGC based on horizontal and diagonal gradient prior principle) operators and LGC-FN (LGC based on a 5 × 5 neighborhood horizontal and diagonal gradient prior principle) operators mentioned below. An LGC-HD operator gives priority to gradients in the horizontal and diagonal directions, which reduces the feature dimension. The formula is shown in (3), where R = 1, P = 6:

(3) $$LGC-HD_{P}^{R}=s\left(g_{1}-g_{3}\right)2^{4}+s\left(g_{4}-g_{5}\right)2^{3}+s\left(g_{6}-g_{8}\right)2^{2}+s\left(g_{1}-g_{8}\right)2^{1}\;+s\left(g_{3}-g_{6}\right)2^{0}$$

#### 2.1.3. The LGC-FN Operator Theory

**Figure 3:**
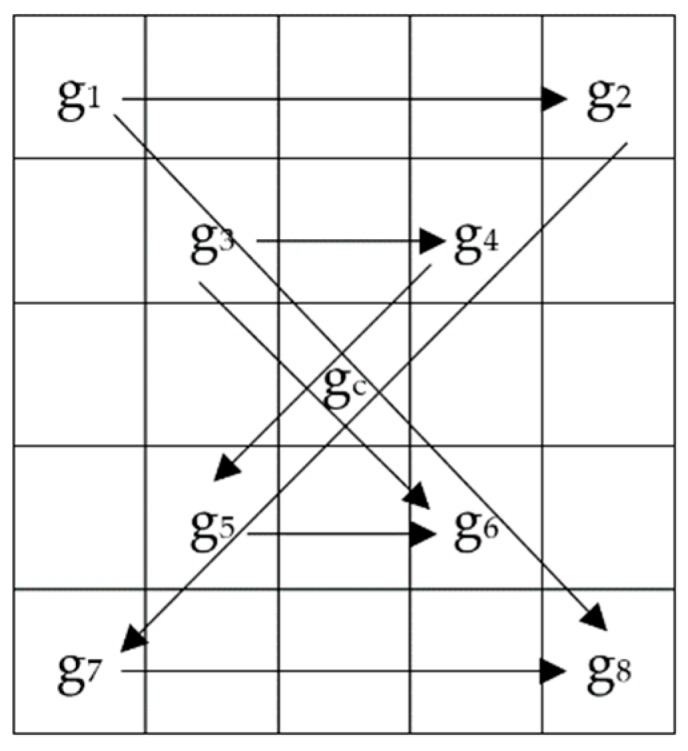
Sample mask for 5 × 5 operators.

The LGC-FN operator expands the neighborhood to a 5 × 5 grid, and calculates the feature values in three directions, horizontal and two diagonal, around the target pixel. The model diagram is shown in Figure 3 and the coding formula is (4). Among them, R = 2, P = 8:

(4) $$\begin{array}{c} LGC-FN_{P}^{R}=s\left(g_{1}-g_{2}\right)2^{7}+s\left(g_{3}-g_{4}\right)2^{6}+s\left(g_{5}-g_{6}\right)2^{5}+s\left(g_{7}-g_{8}\right)2^{4}\\ \;+s\left(g_{1}-g_{8}\right)2^{3}+s\left(g_{3}-g_{6}\right)2^{2}+s\left(g_{2}-g_{7}\right)2^{1}+s\left(g_{4}-g_{5}\right)2^{0} \end{array}$$

#### 2.1.4. The LGC-AD Operator Theory

The LGC-AD operator directly adds the vertical gradient information to the LGC-FN operator. Model diagram refers to Figure 3. The formula is shown in (5).

(5) $$\begin{array}{c} LGC-AD_{P}^{R}=s\left(g_{1}-g_{2}\right)2^{11}+\;s\left(g_{3}-g_{4}\right)2^{10}+\;s\left(g_{5}-g_{6}\right)2^{9}+\;s\left(g_{7}-g_{8}\right)2^{8}\\ +s\left(g_{1}-g_{7}\right)2^{7}+\;s\left(g_{3}-g_{5}\right)2^{6}+\;s\left(g_{4}-g_{6}\right)2^{5}+\;s\left(g_{2}-g_{8}\right)2^{4}\\ +s\left(g_{1}-g_{8}\right)2^{3}+\;s\left(g_{3}-g_{6}\right)2^{2}+\;s\left(g_{2}-g_{7}\right)2^{1}+\;s\left(g_{4}-g_{5}\right)2^{0} \end{array}$$

### 2.2. Proposed Method

**Figure 4:**
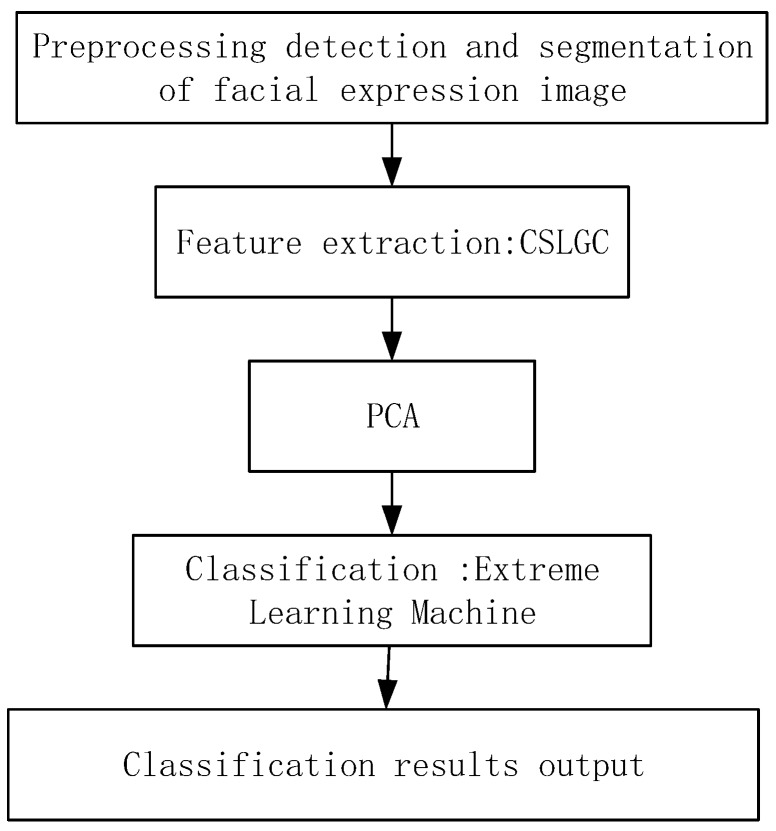
Symmetrical local texture coding-based FER system block diagram.

We propose a balanced and symmetrical local texture coding-based FER system. The structure diagram of this system is shown in Figure 4.

#### 2.2.1. FER Data Characteristics

There are still some problems with the LGC operator and its extensions. LGC operator compares three groups of horizontal and three groups of vertical pixels, but only two groups of diagonal pixels are compared. As a result, the facial feature extraction in the diagonal direction of the gradient information is reduced, which negatively influences on the recognition accuracy.

The LGC-HD operator deletes the gradient information in the vertical direction, reduces the dimension of the feature, and has no significant impact on the recognition accuracy in a 3 × 3 neighborhood. However, the LGD-FN operator extends its neighborhood to a 5 × 5 grid. We believe that the information in the vertical expression cannot be completely ignored. In addition, the correlation between horizontal and central pixels selected by LGC-FN operators is not the strongest, and the characteristics of central pixels cannot be described in more detail. Finally, if this supplementary information is directly included, it leads to higher operational dimensions.

#### 2.2.2. CS-LGC

**Figure 5:**
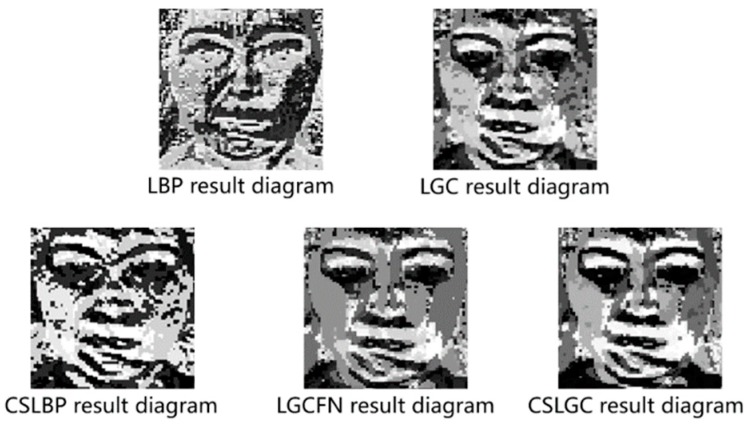
All kinds of algorithm result graphs.

In order to solve these problems, we use the idea of central symmetry, and propose a centrally-symmetric local gradient encoding (CS-LGC) method, which calculates the eigenvalues of the selected pixels in the direction of the target pixels from the horizontal, vertical, 45°, and 135°. The proposed method is as follows; the results of CS-LGC and other operators’ feature extraction are shown in Figure 5:

(6) $$\begin{array}{c} CSLGC_{P}^{R}=s\left(g_{13}\;-\;g_{16}\right)2^{7}+\;s\left(g_{14}\;-\;g_{15}\right)2^{6}+\;s\left(g_{9}\;-\;g_{12}\right)2^{5}+\;s\left(g_{10}\;-\;g_{11}\right)2^{4}\\ +s\left(g_{1}\;-\;g_{8}\right)2^{3}+\;s\left(g_{3}\;-\;g_{6}\right)2^{2}+\;s\left(g_{2}\;-\;g_{7}\right)2^{1}+s\left(g_{4}\;-\;g_{5}\right)2^{0} \end{array}$$

**Figure 6:**
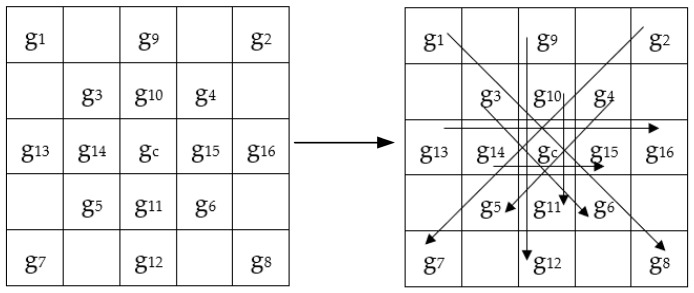
The design of the central symmetric LGC (CS-LGC).

First, we select a 5 × 5 neighborhood and perform the CS-LGC operation on pixels in the 0°, 90°, 45°, and 135° directions, and compare the values of the corresponding location pixels in this order as shown in Figure 6. Then, according to (6), we compute a set of binary strings. Finally, the binary strings are converted into a decimal number to get the final pixel value. Among them, R = 2, P = 8.

**Figure 7:**
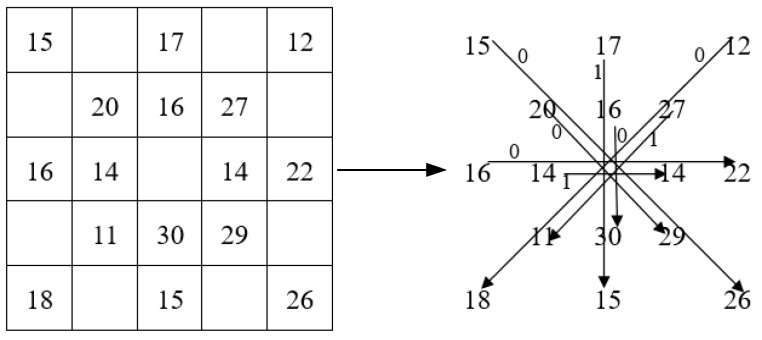
CS-LGC operator operation example.

For example, as shown in Figure 7, we use the CS-LGC operator to calculate the target pixel to get a binary string of 01100001. Converting it to a decimal value of 0 × 128 + 1 × 64 + 1 × 32 + 0 × 16 + 0 × 8 + 0 × 4 + 0 × 2 + 1 × 1 = 97, the feature value of the final target pixel is 97.

#### 2.2.3. CS-LGC Feature Extraction

**Figure 8:**
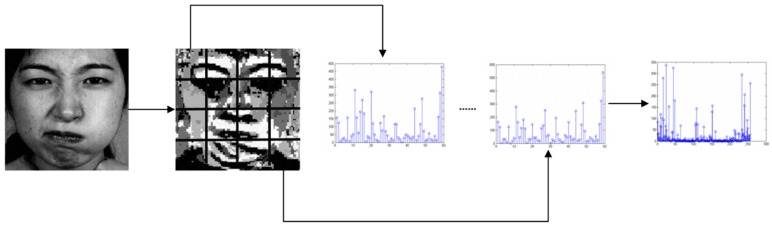
CS-LGC feature extraction process.

Histograms can effectively describe the global features of image texture, but histogram statistics directly on the whole expression image will lose a lot of structural details. Therefore, the expression image is divided into several equal-sized and non-overlapping sub-blocks, and then the histogram of each sub-block area is counted. Finally, the histograms of each sub-block are cascaded, and the cascaded histograms represent the features of the expression image. In this paper, the specific steps of feature extraction process are as follows:

(1) Facial expression image is divided into m × n sub-blocks.
(2) Each sub-block is coded by CS-LGC operator, and the texture image of CS-LGC is obtained.
(3) The CS-LGC histogram of each sub-block is counted.
(4) Cascade the histograms of m × n sub-blocks. The cascaded histogram shows the features of the expression image. The extraction process is shown in Figure 8.

#### 2.2.4. Dimensionality Reduction

Because the dimensions of the CS-LGC feature vector are relatively high, in order to not affect the processing efficiency (in addition to removing redundancy and noise) in the facial expression feature, we use a Principal Component Analysis (PCA) [17] algorithm to reduce the dimensions of the feature matrix. PCA is a common data analysis method. It transforms the original data into a set of linear independent representations of each dimension through a linear transformation, and can be used to extract the main feature components of the data. When the contribution rate is 0.95, the main features extracted by the PCA algorithm can already describe the overall characteristics. At this rate, the characteristic dimension is not large, and the recognition performance is not very different from the performance when the contribution rate is greater than 0.95. Therefore, in the experiment, we select 0.95 as the contribution rate. It can effectively improve the efficiency of the FER system.

#### 2.2.5. Training and Classification

In the experiment, we use the Extreme Learning Machine (ELM) algorithm [18] for training and classification. A SLFNs with *L* hidden nodes is described as follows:

(7) $$f_{L}\left(x\right)=\overset{L}{\underset{i\;=\;1}{\sum }}\beta_{i}G\left(a_{i}\;,b_{i}\;,x\right)=h\left(x\right)\beta$$

where G (*a_i_*, *b_i_*, *x*) is the activation function with the hold parameters *a_i_* and *b_i_*.

Compared with other training algorithms, the ELM algorithm does not need to adjust the input weights and the bias of the implied network neurons, so that the unique solution can be obtained with higher efficiency. Since its initial introduction, the network has shown better performance than traditional BP algorithm and SVM in solving complex pattern recognition problems because of its simple structure, fast speed, and good generalization performance [19]. Therefore, the ELM algorithm is selected for training and classification in this paper.

## 3. Results

In order to evaluate the performance of the proposed operator on facial expression images, we have conducted experiments on the JAFFE database [20] (The database was planned and assembled by Michael Lyons, Miyuki Kamachi, and Jiro Gyoba. The photos were taken at the Psychology Department in Kyushu University Fukuoka, Japan) and the CK+ database [21] (The database was planned and assembled by Patrick Lucey, Jeffrey F. Cohn, Takeo Kanade, Jason Saragih, Zara Ambadar, University of Pittsburgh, Pittsburgh, PA, USA). The experimental software environment consists of MATLAB R2016b (MATLAB is produced by MathWorks, Natick, MA, USA). The hardware environment’s CPU is an Intel (R) Core (TM) i7-7700HQ @ 2.80 GHz; the system’s memory is 8 GB. The operating system was 64-bit Microsoft Windows 10.

### 3.1. Experiments on the CK+ Database

**Figure 9:**
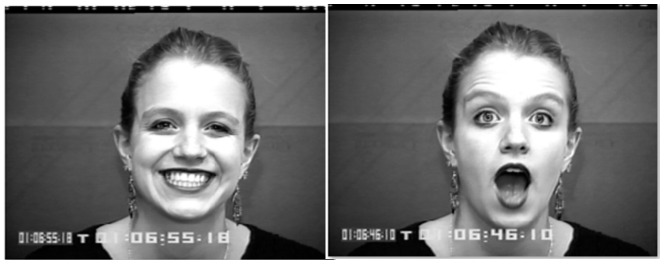
The examples of CK+ face database.

The CK+ database is based on the Cohn-Kanade Dataset and was released in 2010. The database contains 123 subjects, 593 image sequences, and the last frame of each image sequence has the label of action units. The size of each image is 640 × 490. Of the 593 image sequences, there are 327 sequences with emotion. The database contains women, men, Europeans, Africans, and some other people. Everyone has six expressions: angry, disgusted, fearful, happy, sad, and surprised. Some samples in CK+ database are presented in Figure 9.

We have experimented with all six expressions of 20 people in the CK+ library. Each person randomly selected three pictures for each expression. A sample of 360 images has been selected. We detect the face of the original image, and normalize face image to 128 × 128 pixels.

#### 3.1.1. Choose the Best Image Blocking Method

Because the number of sub-images affects the size of the image feature matrix, using image partitioning and a sub-image feature matrix to represent a facial expression image can more fully represent its information. This can increases the effective recognition of features and better reflects the differences of the local features of a facial expression. In order to discuss the influence of image segmentation on the recognition algorithm, the effect of image segmentation on the recognition rate is explored in this paper as follows. When the number of sub-images is too large, the feature matrix becomes very large, requiring substantial computation time. Therefore, in the trial, we select the following blocking methods from the original image: 1 × 2, 1 × 4, 2 × 2, 2 × 4, 4 × 2, and 4 × 4. In this experiment, we select 20 images of each expression for training, and the remaining 40 images for testing. The experimental results are shown in Table 1. From this, we can see that when the block mode is 1 × 4, the recognition rate is the highest, reaching 98.33%. Therefore, we use the 1 × 4 block method in the subsequent experiments. We divide 128 × 128 facial expression images into 4 blocks according to 1 × 4 block method. The histogram size of each region feature image is 1 × 4096.

#### 3.1.2. Compare the Performance of Different Operators

**Figure 10:**
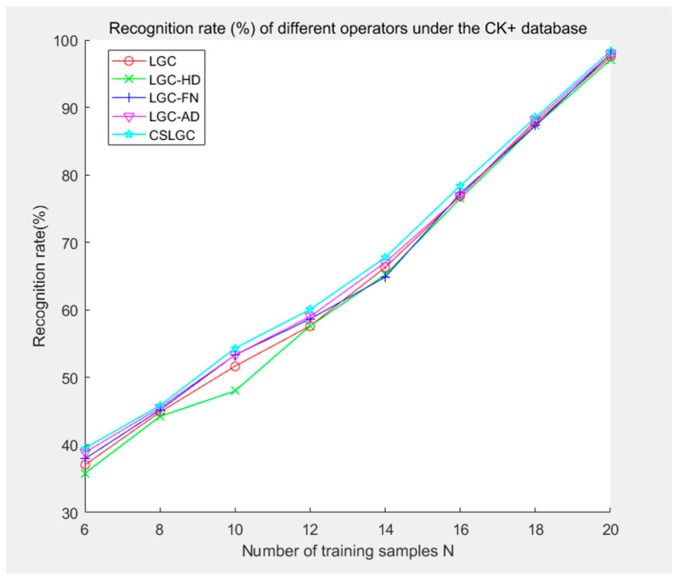
Recognition rate (%) of different operators under the CK+ database.

We select training samples n = 6, 8, 10, 12, 14, 16, 18, 20 for this experiment. We compare the CS-LGC operator with LBP, CSLBP, LGC, LGC-HD, LGC-FN, and LGC-AD operators. The experimental results are shown in Table 2 and Figure 10.

**Figure 11:**
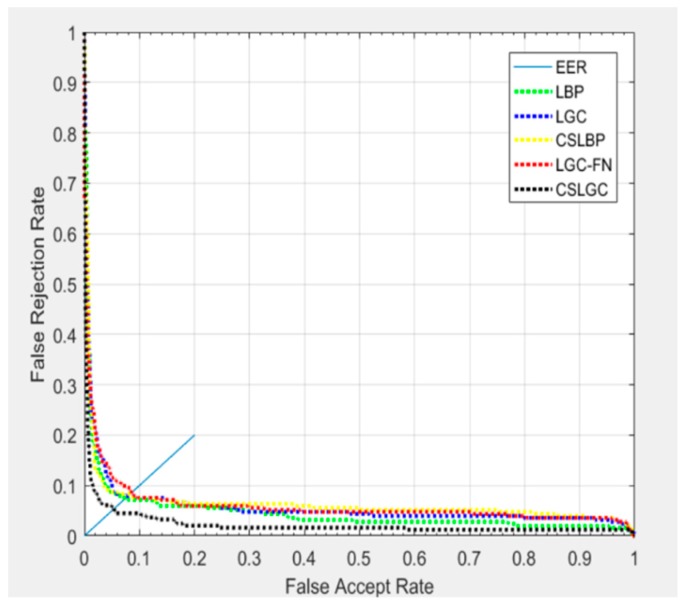
ROC curves comparison of different methods on CK+ database.

As shown in Figure 11, we have plotted the Receiver Operating Characteristic (ROC) curves. In this experiment, we select 20 samples of each expression for training, and the remaining 40 are tested. The curve of the CS-LGC is below the other curves, which means that the CS-LGC method providese the best performance.

### 3.2. Experiments on the JAFFE Database

**Figure 12:**
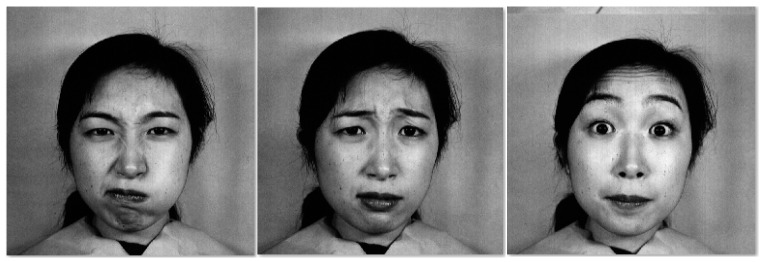
The examples of JAFFE face database [11].

The JAFFE database has a total of 213 emoticons of 10 women; there are seven expressions in the pictures: anger, disgust, fear, happiness, neutral, sadness, and surprise. Each expression has three or four samples. The size of each image is 256 × 256. Some samples in JAFFE database are presented in Figure 12. We first face detection in the original image, and normalize face image to 128 × 128 pixels. We use the 1 × 4 block manner for the experiments. Due to the small amount of data in the JAFFE database, in order to ensure the reliability of the experiment, we use the Leave-p-out Cross Validation method to conduct the experiments.

#### Compare the Performance of Different Operators

**Figure 13:**
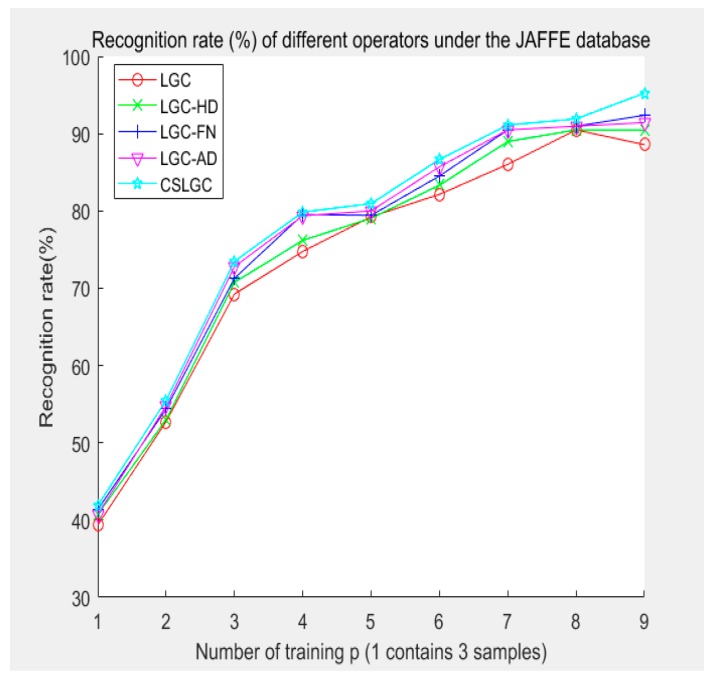
Recognition rate (%) of different operators under the JAFFE database.

In this experiment, three samples of for each of the seven expressions are organized into a collection. Each of these collections is further divided into 10 groups. Next, nine groups of experiments are performed: leave-1-out, leave-2-out, … and leave-9-out. Finally, the average of five, randomly selected experimental results for each group of experiments is used to compute the final recognition rate. We compare the CS-LGC operator with LBP, CSLBP, LGC, LGC-HD, LGC-FN and LGC-AD operators. The experimental results are shown in Table 3 and Figure 13.

**Figure 14:**
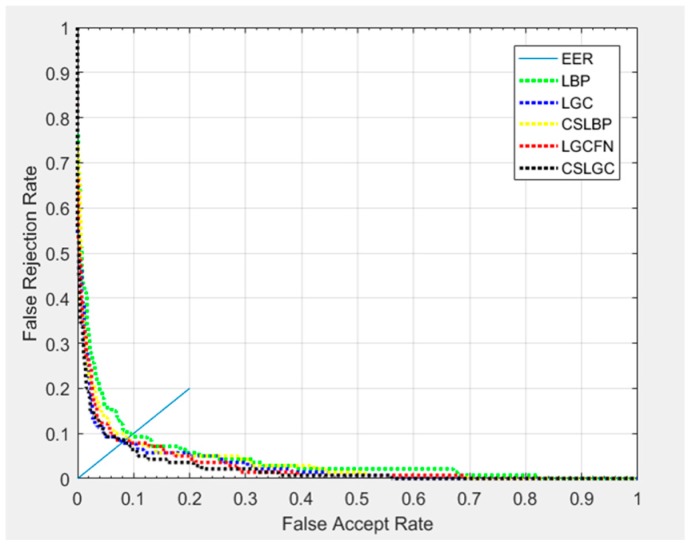
ROC curves comparison of different methods on JAFFE database.

As shown in Figure 14, we have plotted the Receiver Operating Characteristic (ROC) curves. In this experiment, we select 10 samples of each expression for training, and the remaining 20 are tested. Although the CS-LGC curve has some intersection with the other curves, it is still below the other curves, which indicates its strong performance.

### 3.3. The Processing Time of Different Operators

In the experiment, we compare the processing time of the LBP, CSLBP, LGC, LGC-HD, LGC-FN, LGC-AD and CSLBC operators. Table 4 shows the processing time of each operator. The LGC-AD operator has the longest processing time among the derivative LGC operators, which is due to the high feature dimension. CS-LGC operators introduce the idea of centrosymmetric. On the basis of guaranteeing the Omni-directional selection of information, the feature size is reduced and the operation efficiency is improved.

## 4. Discussion

From Table 2 and Table 3, we can see that compared with other operators, the CS-LGC operator has certain advantages in the recognition rate for the CK+ database and the JAFFE database, which we attribute mainly to two factors. First, the LBP operator only considers the relationship between the central pixel and the adjacent pixel, ignoring the correlation between the adjacent pixels. The coding method of the LGC and its extension operators is consistent with the changing trend of human eyes, mouth, and other features. Fully considering the influence of texture changes on expression, the LGC and its extension operators are more suitable for facial expression recognition. Secondly, in the 3 × 3 neighborhood there is little difference in the recognition accuracy between the LGC and LGC-HD operators. Some studies also show that there is less information in the expression of the vertical gradient, but this does not mean that the information in the expression of the vertical gradient can be completely ignored. When the neighborhood is extended to a 5 × 5 grid, the influence of the vertical gradient information on the recognition results increases with the increase of information. However, adding the vertical gradient information directly to the operator increases the feature dimension. The vertical gradient information is added using the principle of centrosymmetric; hence, the CS-LGC operator fully considers the vertical gradient information, the gradient information between centrosymmetric relational pixels, and the relationship between the adjacent pixels. While guaranteeing a lower training dimension, the vertical gradient information is added to improve the recognition rate.

At the same time, we also find that the impact of the CS-LGC operator is more apparent in the JAFFE database, mainly because the facial expression gradient information in this database is rich, and some other restrictions, such as gender, race, and environment, affect together. However, from the overall performance of the operator, introducing the concept of centrosymmetric can extract more uniform and effective gradient information, which is helpful to further study the horizontal, vertical, and two diagonal directions of the neighborhood.

## 5. Conclusions

Overall, the CS-LGC operator has certain advantages in the field of facial expression recognition. Compared with other operators, the CS-LGC operator has better performance and generalization ability; its lower computational cost supports its broad use in the field of facial expression recognition. Compared with deep learning methods, traditional computing approaches offer significant performance advantages, as they are simple and effective. At the same time, we also recognize that the formation of facial expression recognition in practice is a dynamic process. Although this operator is suitable for static facial expression recognition, further research is needed to recognize dynamic expressions in video sequences.

## Figures and Tables

**Table 1:** Recognition rate of different block manner using CS-LGC operator on CK+ database.

Block Manner	1 × 2	1 × 4	2 × 2	2 × 4	4 × 2	4 × 4
Recognition rate (%)	97.78	98.33	97.78	96.11	97.78	96.11

**Table 2:** Recognition rate (%) of LGC-FN, LGC-AD and CS-LGC under the CK+ database.

Number of Training Samples (N)	Operators						
	LBP	CSLBP	LGC	LGC-HD	LGC-FN	LGC-AD	CS-LGC
N = 6	36.11	36.73	37.04	35.80	37.96	38.89	39.51
N = 8	43.91	44.55	44.87	44.23	45.19	45.51	45.83
N = 10	49.00	51.67	51.67	48.00	53.33	53.33	54.33
N = 12	56.25	57.64	57.64	57.64	58.68	59.03	60.07
N = 14	64.86	67.39	66.30	65.22	64.86	67.03	67.75
N = 16	76.14	76.89	76.89	76.52	77.27	76.89	78.41
N = 18	85.71	86.51	87.70	87.30	87.30	88.10	88.49
N = 20	96.47	97.08	97.50	97.08	97.92	97.92	98.33

**Table 3:** Recognition rate (%) of different operators under the JAFFE database.

Number of Training Samples P (One Copy Contains Three Samples)	Operators						
	LBP	CSLBP	LGC	LGC-HD	LGC-FN	LGC-AD	CS-LGC
P = 1	34.92	39.15	39.37	40.74	41.37	40.74	41.90
P = 2	48.10	51.90	52.73	52.98	54.37	54.76	55.48
P = 3	57.82	63.95	69.25	70.75	71.29	72.79	73.47
P = 4	65.24	72.38	74.76	76.19	79.52	79.37	79.84
P = 5	66.48	73.52	79.24	79.05	79.43	80.00	80.95
P = 6	73.09	80.00	82.14	83.33	84.52	85.71	86.66
P = 7	75.23	81.90	86.03	88.98	90.48	90.48	91.11
P = 8	77.14	86.67	90.48	90.48	90.96	90.96	91.91
P = 9	84.76	88.57	88.57	90.48	92.38	91.43	95.24

**Table 4:** The processing time of different algorithms.

Algorithms	LBP	CSLBP	LGC	LGC-HD	LGC-FN	LGC-AD	CS-LGC
Processing Time (s)	0.017	0.019	0.016	0.016	0.014	0.032	0.015
